# Molecularly Imprinting–Aptamer Techniques and Their Applications in Molecular Recognition

**DOI:** 10.3390/bios12080576

**Published:** 2022-07-29

**Authors:** Qingqing Zhou, Zhigang Xu, Zhimin Liu

**Affiliations:** Faculty of Science, Kunming University of Science and Technology, Kunming 650500, China; zhouqingqing678@163.com

**Keywords:** molecularly imprinted polymer, aptamer, molecular recognition

## Abstract

Molecular imprinting–aptamer techniques exhibit the advantages of molecular imprinting and aptamer technology. Hybrids of molecularly imprinted polymer–aptamer (MIP–aptamer) prepared by this technique have higher stability, binding affinity and superior selectivity than conventional molecularly imprinted polymers or aptamers. In recent years, molecular imprinting–aptamer technologies have attracted considerable interest for the selective recognition of target molecules in complex sample matrices and have been used in molecular recognition such as antibiotics, proteins, viruses and pesticides. This review introduced the development of molecular imprinting–aptamer-combining technologies and summarized the mechanism of MIP–aptamer formation. Meanwhile, we discussed the challenges in preparing MIP–aptamer. Finally, we summarized the application of MIP–aptamer to the molecular recognition in disease diagnosis, environmental analysis, food safety and other fields.

## 1. Introduction

The recognition and detection of target molecules in real samples are susceptible to interference from substrates. A variety of naturally occurring biomolecule pairs in biological systems, such as enzyme–substrate and antigen–antibody, have high binding strength and specificity for targets [1] and are thus widely used in the detection of target molecules. However, natural molecular recognition often suffers from low stability, difficult modification, easy denaturation and high cost [2]. Additionally, their use is limited by mild conditions. Therefore, exploring molecular recognition systems with excellent stability and selectivity are important.

Molecular imprinting has attracted much interest among researchers over the past two decades. Molecular imprinting technologies are biomimetic molecular recognition technologies that can synthesize molecularly imprinted polymers (MIPs) with selectivity and sensitive to analytes through chemical reactions. MIPs have highly cross-linked macromolecular structures around template molecules. After template molecules are eventually eluted, imprinted cavities are complementary to template molecules in terms of shape, size and chemical functionality [3,4] and can selectively rebind to templates. MIPs have some advantages, such as low cost, good physicochemical stability, resistance to harsh environments and reusability [5]. Unfortunately, some macromolecules in real samples can be retained on the surfaces of MIPs, resulting in the selective binding sites being blocked that reduce selectivity [6]. Moreover, targets in complex samples cannot be specifically recognized due to interferences by other substances through nonspecific binding.

In recent years, to overcome these issues, many researchers have proposed novel specific molecular recognition technologies based on MIP–aptamer. Combining MIPs with aptamers can better augment their advantages and increase design flexibility. Aptamers are short DNA or RNA oligonucleotide sequences with high affinity and specificity for targets [7]. The binding of aptamers to targets depends on the diverse three-dimensional structures and spatial conformations of single-stranded nucleic acid. Aptamers have the advantages of biocompatibility, wide availability, flexible structure and ease of modification [8] and have high specificity, thereby reducing the nonselective adsorption of MIPs. Combinations of MIPs and aptamers improve the specificity and affinity of polymers to target molecules, and are thus of great research significance.

In this review, we focused on the mechanism of MIP–aptamer and then summarized the fabrication and described their applications in the field of molecular recognition. Finally, we discussed the challenges and future development trends of MIP–aptamer in molecular recognition.

## 2. The Development of Molecularly Imprinted Polymer–Aptamer

MIPs and aptamers, as effective mimetics of antibodies, have similar chemical structures to recognition properties of the natural molecules. Molecular imprinting was first reported by Polyakov’s pioneering work using silicon pore substrates in 1931 [9]. In general, the preparation of MIPs requires the single-template imprints of targeted molecules or similar molecules that can bind to the functional groups of functional monomers through covalent or noncovalent molecular interactions. Template molecules can be removed from prepared polymers, leaving imprinted 3D skeleton cavities for the specific rebinding of target molecules [10,11]. The ability of MIPs to recognize targeted analyte molecules is influenced by many factors, including ionic strength, pH, solution polarity and polymer type and size [12]. Aptamers were first proposed by Andrew Ellingtin and Jack Szostak in 1990 [13] and were screened from synthetic high-capacity single-stranded random oligonucleotide libraries through the systematic evolution of ligands and an exponential enrichment technique (SELEX) [14]. Aptamers may directly bind a wide range of target analyte molecules, such as metal ions [15], small molecules [16,17], biomolecules [18], microorganisms [19] and cells [20]. The complex and unique three-dimensional shapes of aptamers, including loops, hairpins, pseudoknots, bumps, triplets, branches and quadruplexes complementary to the target molecule, are formed by folding through hydrogen bonding between bases within a chain [21,22,23]. The folding of an aptamer into a 3D shape depends on many factors, such as temperature and salt concentration. Owing to the dynamic structures of aptamers, the binding of aptamers to targets is unstable. Furthermore, they are susceptible to thermal, chemical and enzymatic degradation. Their stability can be increased by introducing a functional molecule at the 5′ or 3′ end.

MIPs and aptamers have been used individually to recognize various molecules in complex matrices. However, given that MIPs and aptamers have their own advantages and disadvantages, combining MIPs and aptamers may be an ideal solution for synthesizing adsorbent materials with improved properties and desirable features. The comparison of the features of MIP, aptamer and MIP–aptamer was listed in Table 1. In 2013, Spivak et al. prepared the first hydrogel based on MIP–aptamer for the selective recognition of target proteins with high affinity and stability [24]. Introducing aptamers as functional monomers into MIPs can increase binding affinity and specificity. The polymer matrix protects DNA aptamer strands from nuclease enzyme degradation, improving aptamer stability. A new way for molecular specific recognition is opened. Synthesized MIPs can be formed into various morphological structures, such as bulk materials, nanomaterials, nanocomposites and thin films. MIP–aptamers based on substrate such as CdSe quantum dots, metal–organic framework and upconversion nanoparticles have been intensely investigated as promising analytical devices that will further broaden the range of applications of MIP–aptamers. MIP–aptamers also have been used in molecular recognition and detection in the fields of environmental analysis, pathogen detection, food safety and other fields. The applications of the most interesting MIP–aptamers since 2013 are shown in Table 2. MIP–aptamers are applied to the analysis of target in the complex matrix with a wide linear range and a low detection limit.

## 3. Synthesis of MIP–Aptamer

**Figure 1:**
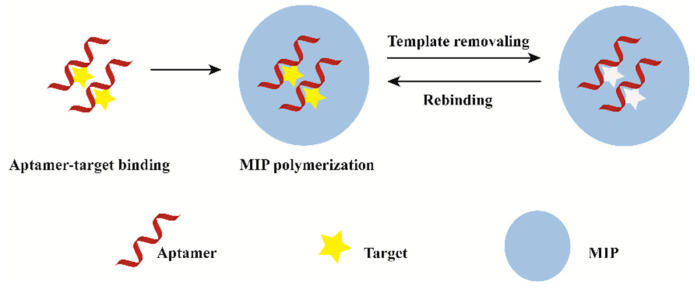
General description of the synthesis process of MIP–aptamer.

Hybridized MIP–aptamer exhibit many advantages over MIPs or aptamers, which had been widely used in molecular recognition. Different methods for preparing MIP–aptamer have been reported in the literature. The commonly used method is to mix the target aptamer with target molecules to form aptamer–target molecule complexes. Then, functional monomer, polymerization solvents, cross-linkers and initiators are added for the preparation of polymer-covered aptamer–target molecule complexes. Finally, imprinted molecules are eluted. The process of preparation is shown in Figure 1. After the correct folding of aptamers with targets, functional monomers are polymerized around the aptamer–target molecule complexes via molecular imprinting technique. Meanwhile, functional monomers interact with the remaining functional groups of targets to form co-recognition substrates. Polymers synthesized by this method have good recognition specificity. The difference of synthetic methods is the order in which the aptamers are added. Firstly, traditional functional monomer and template molecule pre-polymerized for hours. Then, an aptamer was added for polymerization. This operation may result in insufficient contact between the aptamer and the template molecule. As a result, there are few aptamer-specific recognition sites. Furthermore, computational modeling can improve the aptamer selection process during synthesis.

## 4. Application of MIP–Aptamer in the Molecular Recognition of Complex Samples

### 4.1. Biomarkers

Biomarkers are available for measurement and can reflect a physiopathological process or therapeutic effect. The availability of biomarkers not only helps to investigate the pathogenesis of complex diseases, but also contributes to the screening, treatment and prognosis of diseases in clinical practice.

**Figure 2:**
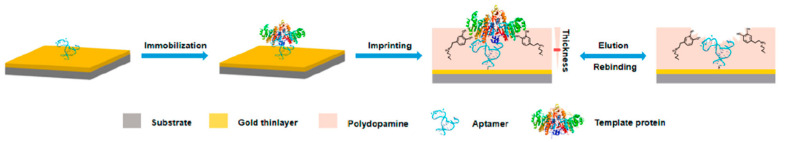
Schematic illustration of the principle and procedure for preparing aptamer–MIP hybrids. Reproduced with permission from [26]. Copyright American Chemical Society, 2019.

Proteins, as a kind of disease marker, have complex and diverse structures. Most proteins have extremely low content in organisms and are thus difficult to recognize and analyze. Therefore, the selective recognition and separation of proteins in complex biological samples are of great significance. To increase the specificity and affinity for glycoprotein alkaline phosphatase in the sera of patients with hepatocarcinoma, Wei Li et al. [26] presented a novel approach based on MIP–aptamer. First, aptamers bound to glycoprotein alkaline phosphatase with relatively weak affinity and specificity as the imprinting recognition safeguard. Then, the surface-oriented imprinting of dopamine in water self-polymerization formed a thin polydopamine layer that covered the template. Finally, MIP–aptamer was obtained by removing the template from the polymer, as shown in Figure 2. The polymerization of polydopamine occurred in a pure water system, which helped to maintain the original conformation of the target molecule and facilitate the rebinding of the target molecule to MIP–aptamer. MIP–aptamer showed cross-reactivity of 3.2–5.6% and satisfactory sensitivity with low limit of detection (6.2 × 10^−12^ M).

Wang et al. utilized magnetic microspheres as substrates [30] and MIP–aptamer as double-recognition layers to fabricate nanoprobes. First, magnetic nanoparticles functionalized by hydrosulfuryl aptamers (Mag Au@SH-aptamers) were synthesized, which captured alpha-fetoprotein. The aptamers were integrated into magnetic nanoparticles through Au–S bonds, and SiO_2_ was used as the imprinted layer. Finally, high-throughput matrix-assisted laser desorption/ionization time of flight mass spectrometry, a rapid and highly efficient method for recognizing and analyzing protein biomarkers, was performed. The MIP–aptamer was immobilized on the surface of magnetic nanoparticles. MIP–aptamer could be quickly separated from the extraction solution by using a magnet. Mag Au@SH-aptamer@MIP exhibited high recognition ability toward alpha-fetoprotein and successfully distinguished protein biomarkers in healthy sera and sera of patients with hepatic carcinoma.

Sullivan et al. [33] synthesized MIP–aptamer polymers to recognize trypsin. Aptamers were used as the recognition elements of polymers, and the chemical structures of the aptamers were slightly modified. The synergistic effects of the MIPs and aptamers contributed to the highly specific recognition ability of the MIP–aptamer to trypsin, and binding affinity was higher than that of conventional MIPs. The limit of detection of the MIP–aptamer was over half (2.4 nm) that of MIPs (4.1 nm). In addition, Roushani et al. [55] detected ultra-trace trypsin in human serum and urine by using a MIP–aptamer. Satisfactory results were obtained with recoveries ranging from 94.0 to 114.0%.

Mokhtari et al. [29] fabricated MIP–aptamer to capture cardiac troponin I for imprinting recognition. First, amino terminus cardiac troponin I aptamers was bound to the surfaces of COOH-ZnO nanoparticle-modified GCEs through covalent immobilization, and then methylene blue functional monomers were electropolymerized around the cardiac troponin–aptamer complexes. After the removal of cardiac troponin, cavities for the recognition of cardiac troponin I in human serum formed. The detection limit of the MIP–aptamer was 2.61 × 10^−5^ μg/mL, with recovery rates of 93.40–114.28% and quantification limit of 2.90 × 10^−5^ μg/mL.

Krishnan et al. [35] developed a biomimetic biosensor to detect human clotting factor IX protein (FIX) for early detection of bleeding disorders by using the MIP–aptamer strategy. The MIP–aptamer sensors were more sensitive than conventional aptamer sensors. Moreover, integrating carbon nanohorn, gold nanourchin and MIP–aptamer as hybrid materials could improve the recognition ability [35]. In addition, the MIP–aptamer was also used for the identification of biomarker exosomes in liquid biopsies [36]. The method had a low detection limit (2.27 μmol/L) and a good recovery (104.17%).

In addition, the recognition of other biomarkers such as cytochrome C [25], amyloid-β oligomers [31], dopamine [34], bovine serum albumin [56], prostate [57], scortisol [58] and thrombin [27,32] were reported based on MIP–aptamer dual recognition approach.

### 4.2. Pharmaceutical Analysis

Antibiotics are commonly used to treat and prevent bacterial infections. However, they can be ingested through foodstuff because of their persistence in animals. The adverse effects of antibiotics include allergic reactions and liver and kidney damage and can pollute the water environment. The use of antibiotics and the detection of residues in food has received considerable interest [59,60,61]. Materials that have dual recognition functions and are based on MIP–aptamer have been used in the recognition and analytical detection of antibiotics in real samples.

Liu et al. prepared hybrid probes based on the synergism recognition of MIP–aptamer grafted on upconversion nanoparticles for enrofloxacin recognition [38]. In the first step, biotinylated enrofloxacin aptamers were immobilized on the surfaces of upconversion nanoparticles to entrap enrofloxacin, which was the first imprinting recognition safeguard. Then, while interacting with the residual functional groups of the enrofloxacin, methacrylic acid monomers were polymerized around the enrofloxacin–aptamer complexes with a molecular imprinting technology after the aptamers correctly folded on the target enrofloxacin molecules. The removal of enrofloxacin from polymers formed simultaneous molecular-recognition-imprinted cavities. The MIP–aptamer was used in the recognition of enrofloxacin in the different real samples of fish, showing a detection limit of 0.04 ng/mL and recovery rates between 87.05% and 96.24%. Upconversion nanomaterials have unique luminescence properties, low toxicity, high chemical stability, long lifespan and deep penetration depth in living tissues. They are widely used in biological imaging [38]. MIP–aptamers based on upconversion nanomaterials are also applied in bioimaging.

**Figure 3:**
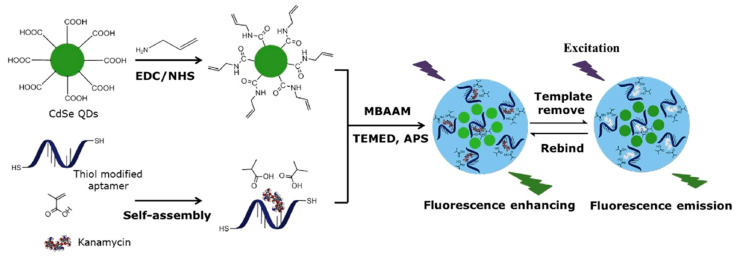
Schematic illustration of the preparation and recognition process for fluorescent aptamer functionalized molecularly imprinted polymers. Reproduced with permission from [42]. Copyright Elsevier, 2018.

A large number of biosensors based on MIP–aptamer have been used to recognize and detect kanamycin. Geng et al. [39] prepared a novel double-recognition fluorescent MIP–aptamer for the high-specificity recognition of kanamycin. The process of preparation is shown in Figure 3. CdSe quantum dots are used as support, thiol-modified kanamycin aptamers and methacrylic acid are used as functional monomers, and kanamycin is used as the template for surface imprinting in aqueous solutions. Aptamers are fixed in polymer matrices through thiol-ene click reactions. The application of thiol-ene click reaction made the polymerization procedure more convenient and efficient. Synergistic interactions between aptamers and methacrylic acid apparently improve the specificity and affinity of MIP–aptamer for kanamycin in food, water and biological samples. The fluorescence intensities of MIP–aptamer exhibit good linear correlation at a concentration of 0.05–10.0 μg/mL, and the detection limit is 0.013 μg/mL. Moreover, Bi at el. proposed a highly specific and sensitive electrochemical method for recognizing and analyzing kanamycin based on the MIP–aptamer [42]. Beta-cyclodextrin is the most common supramolecular host compound, which has a hollow truncated cone structure with a hydrophobic cavity and hydrophilic rims. It can form host–guest clathrates with various molecules, which provide more favorable interactions for the adsorption of target analytes by polymer materials in aqueous media. The use of beta-cyclodextrin increased the adsorption capacity of the MIP–aptamer to the target analytes. The MIP–aptamers showed a good linear relationship in a range of 10–500 nM of kanamycin concentration and a detection limit of 1.87 nM. They have been used successfully to detect kanamycin in spiked milk.

MIP–aptamer has been used in tetracycline analysis. Rad et al. [40] introduced a novel material for sensing tetracycline. First, aptamer–tetracycline complexes were obtained by mixing tetracycline solution with tetracycline aptamer solution. The complexes were immobilized on the surfaces of glassy carbon electrodes (GCEs) modified with gold nanoparticles (AuNPs). Dopamine was then electropolymerized onto the modified GCEs to capture the aptamer–tetracycline complexes. Finally, the tetracycline templet was eluted with an ethanol–acetic acid mixture (95:5, *v*/*v*) to form cavities. AuNPs not only have good conductivity, but also have a large specific surface area. The addition of AuNPs to the sensor can increase the surface area to capture more aptamers and it is more conducive to the specific capability of MIP–aptamer. The novel MIP–aptamer sensor was used successfully in recognizing tetracycline in spiked milk samples, with recovery rates of 94.9–106.2% and an extremely low detection limit of 1.4 × 10^−4^ nM.

Shuhuai Li et al. [37] combined lincomycin aptamers tagged with carbon dots, lincomycin and o-aminophenol on electrodes functionalized by gold-nanoparticle-modified graphene to synthesize carbon dots–MIP–aptamer through electropolymerization. The carbon dots–MIP–aptamer exhibited high selectivity and affinity for lincomycin and was successfully used to recognition lincomycin residuals in meat samples, showing satisfactory results and recovery rates of 89.9–104.5%.

Moreover, MIP–aptamers have been used to recognize ochratoxin A [28], chloramphenicol [41] and moxifloxacin [43].

### 4.3. Pathogen Detection

Viruses are the smallest known microorganisms, with diameters of approximately 20–400 nm [62,63], posing a major threat to humans, agriculture and ecosystems. New viruses emerge every year, and viral diseases have become major global health problems. Owing to the complex surface structures of viruses and their similarities, the recognition of viruses is a major area of interest in many fields, including biomedicine, environmental science and biosecurity. It is of crucial importance to the highly selective and efficient recognition, detection and differentiation of multiple viruses.

Hepatitis C virus (HCV) infection causes chronic liver diseases, which is a global public health problem. Ghanbari et al. [44] grafted MIP–aptamer onto multiwalled carbon nanotube–chitosan nanocomposites for the recognition of HCV core antigen in human serum samples. The MIP–aptamer based on the electropolymerization of dopamine around the HCV cores of antigen aptamer complexes on GCEs modified with multiwalled carbon nanotube–chitosan nanocomposites modified were attained, and the MIP–aptamer showed specificity and high sensitivity for the identification of the hepatitis C virus core antigen. The MIP–aptamer was immobilized on the surface of the multiwalled carbon nanotube–chitosan nanocomposite. The recognition properties of MIP–aptamer were then improved for the high surface area of carbon nanotubes and abundant hydroxyl and amine groups of chitosan. Satisfactory results were obtained; the linear range was from 5.0 fg/mL to 1.0 pg/mL, and the detection limit was 1.67 fg/mL. Furthermore, their study provided theoretical support for the application of MIP–aptamer recognition target molecules in complex matrices.

Hepatitis B virus infection is one of the most dangerous pathogens in human health today. Highly selective detection of the hepatitis B virus is essential. Wang et al. constructed a novel sandwich resonance light-scattering sensor for the hepatitis B virus and based it on MIP–aptamer [45]. Carbon spheres with a large number of -COOH and -OH groups were used as carriers for the preparation of the first recognition probes. Then, aptamers were modified on the surfaces of the spheres for the preparation of the second recognition probes. The introduction of aptamers to the MIP sandwich structures greatly improved the specificity and showed satisfactory selectivity and sensitivity. The addition of tetraethoxysilane provided a stable and controlled thickness of the embossed layer, thus improving the performance of the MIP–aptamer. The imprinting factor was as high as 7.56, and the detection limit was as low as 0.011 nM. The recovery rates were 88.0% and 115.0%.

**Figure 4:**
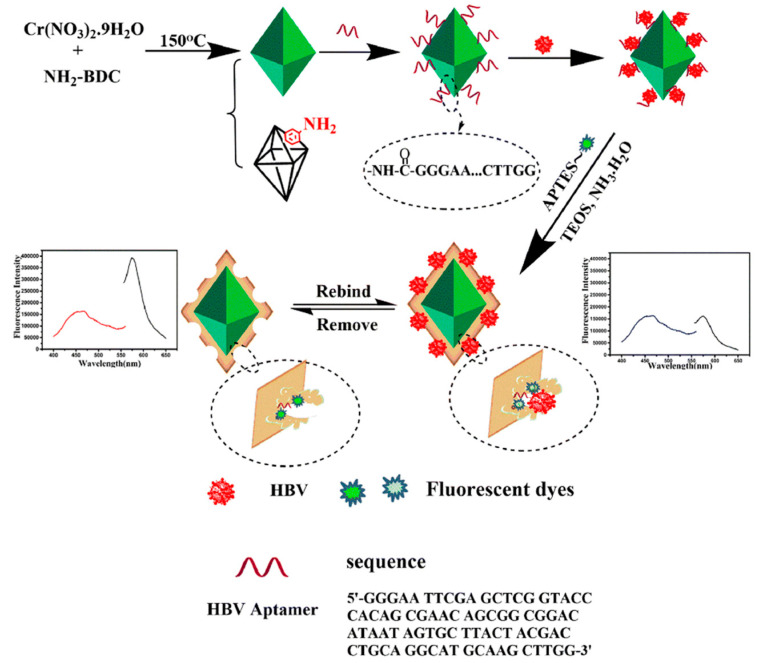
Construction and detection principle of MIP–aptamer. Reproduced with permission from [46]. Copyright Springer, 2021.

Wang et al. [46] used ratiometric metal–organic framework MIL-101-NH_2_ with sufficient imprinting sites as a polymer carrier of MIP–aptamer for the specific recognition of the hepatitis B virus, as shown in Figure 4. The hepatitis B virus aptamer was introduced to the MIL-101-NH_2_ surface through an amide reaction, and then the surfaces were imprinted by tetraethyl silicate self-polymerization for the recognition of hepatitis B and imprinted polymeric cavities and aptamer interaction. Finally, ratiometry fluorometry was performed, and MIL-101-NH_2_ and rhodamine B were used as the reference fluorescent and change signals for hepatitis B virus recognition in a real human blood sample. The metal–organic framework MIL-101-NH_2_ with large specific surface area served as a substrate of MIP–aptamer that provided enough imprinting sites. The MIP–aptamer sensor apparently improved affinity and specificity towards the hepatitis B virus with an imprinting factor of 5.72, a detection limit of 1.8 pmol/L and recoveries between 85.0 and 101%.

Pseudomonas aeruginosa (P. aeruginosa) can cause a long-term chronic disease, and low concentrations of P. aeruginosa can cause a serious infectious bacterium. Sarabaegi et al. [47] designed an electrochemical sensor for the quantitative analysis of P. aeruginosa using MIP–aptamer. The polymeric substrates maintained the aptamer stability and selectivity. Moreover, the MIP–aptamer with many pores was conducive to P. aeruginosa adsorption. Satisfactory results were found in determining P. aeruginosa in blood samples with low detection limit of 1 CFU·m^−1^.

### 4.4. Environmental Analysis

**Figure 5:**
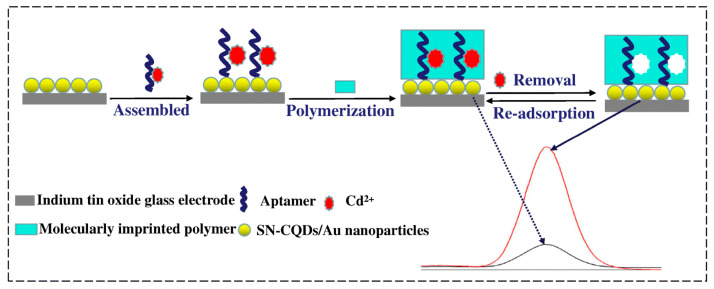
Fabrication of the fluorescence MIP–aptamer sensor for Cd^2+^ detection. Reproduced with permission from [67]. Copyright Springer, 2019.

Cadmium is harmful to the environment and human health, highly toxic, and carcinogenic. Cadmium bioaccumulation in organs, mainly the kidneys, liver and lungs, can cause significant damage to human health and lead to kidney and reproductive dysfunction, osteoporosis and other diseases [64,65]. The World Health Organization has established a maximum contamination level of 5 µg/L for cadmium in drinking water [66]. Therefore, the monitoring of chromium contaminants in aqueous environments remains a challenge in the field environmental chemistry. One of the most promising applications of MIP–aptamer is cadmium (II) recognition [67] (Figure 5). Aptamer–Cd^2+^ complexes were immobilized on carbon quantum dots (codoped with sulfur and nitrogen) and gold-nanoparticle-modified indium tin oxide glass electrode via Au-S bond. Through ultraviolet irradiation-induced polymerization, MIP–aptamer was prepared on the surface of the modified indium tin oxide glass electrode, and L-alanine and N-hydroxysuccinimide were used as a functional monomer and cross-linking agent, respectively. After Cd^2+^ removal, the polymer contained the recognition sites of Cd^2+^ and was successfully applied to the recognition of Cd^2+^ in water, soil and vegetables, showing a detection limit of 1.2 × 10^−12^ mol/L.

Moreover, MIP–aptamer has also been used to recognize environmental pollutants, such as trinitrotoluene [48] and urea [51].

### 4.5. Food Safety

Melamine is a small-molecule organic chemical material widely used in plastics, pigments, fertilizers, adhesives and flame retardants [68]. Its nitrogen content (66.7%) is considerably higher than that of common protein nitrogen (16%) and is thus often added illegally to dairy products, food and pet feed [69]. However, excessive intake of melamine can cause serious damage to the urinary and reproductive systems, leading to bladder stones, kidney failure, bladder cancer and even death in humans and animals [70]. Yu et al. [52] developed a sensor based on MIP–aptamer for the highly specific recognition of melamine. AuNPs were synthesized through the simple reduction of sodium citrate. MIPs with specific recognition sites formed through the electropolymerization of dopamine with polythymine aptamers as functional monomers and melamine as template molecule. The MIP–aptamer showed satisfactory selectivity and sensitivity, a linear relationship between 10^−12^ and 10^−4^ mol/L for melamine detection in milk samples, and detection limit of 6.7 × 10^−13^ mol/L.

Aflatoxins are carcinogenic and mutagenic. Excessive aflatoxin in food poses a huge threat to people’s health and safety. Roushani et al. [53] designed a biosensor based on MIP-amtamer synergistic identification for the detection of aflatoxin B1 (AFB1) in milk. The detection limit of the method was 12.0 pg/L. Modification of the aptamer sequences with amino increased its interaction with the substrate. Cu_2_O, with large specific surface area and high adsorption capacity, was modified on the GCE surface to increase the loading of aptamers. The proposed method can be extended to other target molecules by replacing their aptamer sequences of other target molecules.

Pesticides have high acute toxicity and are used extensively not only in agriculture, forestry, and horticulture but also in domestic applications. Residues at trace levels can cause long-term effects on the environment and human life [71]. A strategy for planning pesticide chlorpyrifos recognition based on MIP–aptamer has been developed and has shown recognition properties superior to that of aptamers or traditional MIPs [50]. First, AuNR was used as an attractive substrate for covalent MIP–aptamer immobilization. Then, GCE was carefully polished with alumina powder. AuNR solution was added dropwise to the polished GCE surface for the preparation of AuNR/GCE composite in the complex solution of the aptamer target. The MIP–aptamer was electropolymerized AuNR/GCE. Under optimal conditions, the detection limit of MIP–aptamer was 0.35 × 10^−6^ nM, and the recovery rate ranged from 97.56% to 103.2%, lower than the rates of previously reported methods.

**Figure 6:**
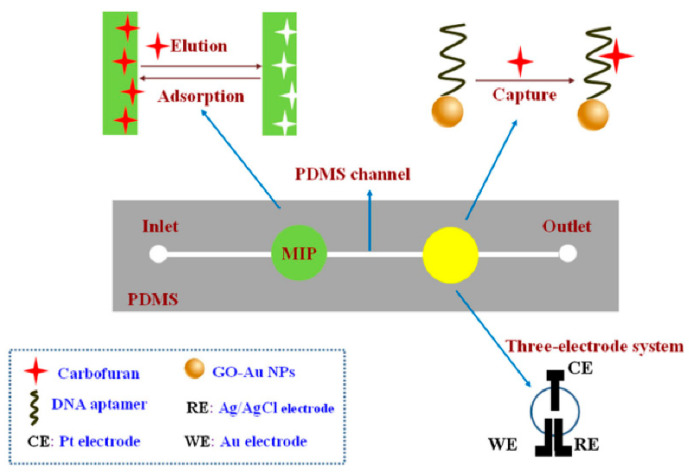
Fabrication of MIP–aptamer microfluidic chip for the detection of carbofuran. Reproduced with permission from [49]. Copyright Springer, 2018.

Moreover, Li et al. [49] combined MIPs and aptamers to prepare an electrochemical microfluidic chip for identifying carbofurans in cabbage, pepper, lettuce, tomato, apple, banana, orange and watermelon, as shown in Figure 6. Carbofuran was transported to MIPs and captured at the recognition site in the channel. Then, carbofuran was eluted with carbinol-acetic acid, transported to the next testing position, and captured again by the aptamer. Finally, it was detected by differential pulse voltammetry. The dual recognition of MIP–aptamer resulted in high selectivity and showed recovery rates between 89.6% and 110.4% and a detection limit of 6.7 × 10^−11^ mol/L.

### 4.6. Other Applications

MIP–aptamer has been successfully used to detect various target analytes in complex matrices. Histamine is a biogenic amine that plays an important role in a variety of pathophysiological processes [72]. The ingestion of high levels of histamine may cause a range of allergic inflammatory diseases and cause cardiac arrest [73,74]. Dual-recognition MIP–aptamer based on carboxylated carbon nanotubes decorated by gold nanoparticles (AuNPs/cCNTs/GCE) for the highly selective and sensitive determination of histamine in different matrices has been established [54]. Histamine–aptamer complexes were pipetted on the surface of AuNPs/cCNTs/GCE, and the resulting histamine–aptamer/AuNPs/cCNTs/GCE covalently bonded to gold nanoparticles through strong Au-S covalent bonds. Histamine was dropped onto the surface of the modified electrode to impregnate any free aptamer. MIP–aptamer/AuNPs/cCNTs/GCE was formed by the electropolymerization of O-phenylenediamine at the surface of aptamer/AuNPs/cCNTs/GCE. Finally, MIP–aptamer/AuNPs/cCNTs/GCE was obtained through the elution of histamine from cavities with acetonitrile/water solution (5:1, *v*/*v*). The recovery rates of MIP–aptamer/AuNPs/cCNTs/GCE were between 95.3% and 104.4%, exhibiting good sensitivity and selectivity toward histamine in melamine analysis in real samples of human blood plasma and canned tuna fish. Finally, MIP–aptamer was used in the analysis of adenosine target molecules [75,76].

## 5. Conclusions and Future Perspectives

MIP–aptamer combines the merits of MIPs and aptamers, showing great promise in molecular recognition. The affinity and specificity of MIP–aptamer significantly improved, lowering the detection limit of the target analyte while improving the stability of target molecule recognition in complex samples. It has broad application prospects in the analytical detection of complex matrices. Unfortunately, small molecules as template molecules may result in fewer recognition sites in the MIP–aptamer, which would be a disadvantage for target-molecule recognition. Usually, aptamers have a long-chain structure. Macromolecules, which have more complex molecular structures and functional groups, interact easily with aptamers. This factor may limit MIP–aptamer application in small-molecule identification. MIP–aptamer can be prepared in a number of ways. Many studies have shown that a single modified aptamer cannot be effectively immobilized into polymers, and making aptamer chains very flexible possibly leads to cross-reactivity and low imprinting factor. Different DNA sequences have different binding properties in MIPs. Multivalent or polydentate aptamers, which have multiple binding sites, may enable aptamers to be effectively immobilized on polymers. Thus, a reasonable DNA sequence design is essential to the detection of target molecules without known ligands. Furthermore, mixed aptamers should also be boldly introduced into the preparation of MIP–aptamers to obtain higher affinity. At the same time, the development of MIP–aptamer for larger targets meets a major challenge due to the lack of available water-soluble functional monomers. Future research should overcome the shortcomings of current experimental synthesis methods and simplify the preparation. This proposed strategy can be easily extended and has potential applications in bioanalysis. Computer modeling will further reduce the experimental optimization work, improve the performance of MIP–aptamer, and ultimately improve the selectivity, sensitivity and stability of recognition.

## Figures and Tables

**Table 1:** Comparison of MIP, aptamer and MIP-aptamer.

Properties	MIP	Aptamer	MIP–Aptamer
Sensitivity	Low	Medium	Ultrahigh
Selectivity	Medium	High	Ultrahigh
Affinity	Low	High	High
Stability	High	Medium	High

**Table 2:** Applications of MIP–aptamer during 2013–2022.

Analyte	Sample	Method	Linearity Range	LOD	Year	Ref.
Proteins, Thrombin and PDGF-*ββ*	urine, tears	Visual detection	-	-	2013	[24]
Cytochrome C	urine, serum	Fluorescence	0.20–2.00 μM	0.054 μM	2018	[25]
Glycoprotein alkaline phosphatase	human serum	Plasmonic immunosandwich assay	-	-	2019	[26]
Thrombin	bovine blood	Electrochemical	2.5 × 10^−9^–1.3 × 10^−6^ mg/mL	1.6 × 10^−10^ mg/mL	2019	[27]
Ochratoxin A	beer	High-performance liquid chromatography-fluorescence	0.05–1.00 ng/mL	0.07 ng/mL	2020	[28]
Cardiac Troponin I	human serum	Voltammetric	0.50–3.3 × 10^5^ pM	1.04 pM	2020	[29]
Alpha-fetoprotein	human serum	Matrix-assisted laser desorption/ionization time-of-flight mass spectrometry	20–1000 ng/mL	0.5 ng/mL	2020	[30]
Amyloid-*β* oligomer	human serum	Electrochemical	5 pg/mL to 10 ng/ mL	1.22 pg/mL	2020	[31]
Thrombin	serum	Colorimetric	1.08 × 10^−10^–2.7 × 10^−5^ mol/L	2.7 × 10^−11^ mol/L	2021	[32]
Trypsin	blood human serum and urine	In situ electropolymerization	1–90 pg/mL	0.75 pg/mL	2022	[33]
Prostate specific antigen	human serum	Electrochemical	100 pg/mL–100 ng/mL	1 pg/mL	2016	[16]
Dopamine	serum	Electrochemical	5.0 × 10^−8^–1.0 × 10^−5^ mol/L	4.7 × 10^−8^ mol/L	2021	[34]
Factor IX protein	human plasma serum	Electrochemical	0.8 fM to 0.8 nM	40 fM	2022	[35]
Exosomes	serum	Fluorescence	1.19 × 10^−6^–4.76 ×10^−5^ mol/L	2.27 × 10^−6^ mol/L	2022	[36]
Lincomycin	meat	Electrochemical	5.0 × 10^−12^–1.0 × 10^−9^ mol/L	1.6 × 10^−13^ mol/L	2017	[37]
Enrofloxacin	fish	Fluorescence	-	0.04 ng/mL	2017	[38]
Kanamycin	water, milk and urine	Fluorescence	8.6 × 10^−8^–1.7 × 10^−5^ mol/L	2.2 × 10^−8^ mol/L	2018	[39]
Tetracycline	milk	Electrochemical	5× 10^−4^–1000 nM	1.4 × 10^−4^ nM	2019	[40]
Chloramphenicol	milk	Electrochemical	1.0 pM to 1.0 nM	0.3 pM	2019	[41]
Kanamycin	milk, tap, artesian groundwater	Electrochemical	10.00–500.00 nM	1.87 nM	2020	[42]
Moxifloxacin		Electrochemical	0.001–1 µM	0.51 nM	2021	[43]
Hepatitis C virus	human serum	Electrochemical	5.0 fg/mL–1.0 pg/mL	1.67 fg/mL	2018	[44]
Hepatitis B virus	human serum	resonance light scattering	0.04–0.1 nmol/L	0.011 nmol/L	2021	[45]
Hepatitis B virus	human blood	Fluorescence	10–3500 pmol/L	1.8 pmol/L	2021	[46]
Pseudomonas aeruginosa	blood	Electrochemical	101 to107 CFU/mL	1 CFU/mL	2021	[47]
Trinitrotoluene	soil, river water	Electrochemical	0.01 fM to 1.5 μM	3.5 × 10^−9^ nmol/L	2017	[48]
Carbofuran	fruit, vegetable	Electrochemical	0.2–50 nM	67 pM	2018	[49]
Chlorpyrifos	apples, lettuce	Electrochemical	1 × 10^−6^–400 × 10^−6^ nM	0.35 fM	2018	[50]
Urea	soil, water	Impedance spectroscopy	0.005–500 nM	900 fM	2019	[51]
Melamine	milk	Electrochemical	10^−12^–10^−4^ mol/L	6.7 × 10^−13^ mol/L	2021	[52]
Aflatoxin B1	milk	Electrochemical	50.0 pg/L to 3.5 ng/L	12.0 pg/L	2022	[53]
Histamine	human blood plasma, canned tuna fish	Differential pulse voltammetry and electrochemical impedance spectroscopy	0.46–35 nmol/L 0.35–35 nmol/L	0.15 nmol/L and 0.11 nmol/L	2020	[54]

## Data Availability

Not applicable.
